# Supplementary material for: A Sensitive and Specific Monoclonal Antibody Based Enzyme-Linked Immunosorbent Assay for the Rapid Detection of Pretilachlor in Grains and the Environment
Source: Foods. 2023 Dec 19;13(1):12. doi: 10.3390/foods13010012 (PMC10777945; doi:10.3390/foods13010012)
Supplement: Supplementary file 1 [file foods-13-00012-s001.zip › foods-2756521-supplementary.pdf]

Article

A Sensitive and Specific Monoclonal Antibody Based Enzyme-Linked Immunosorbent Assay for the Rapid Detection of Pretilachlor in Grains and the Environment

Linwei Zhang [1,†], Xiaoyang Yin [1,†], Hongfei Yang [1], Hao Wen [1], Shiyun Han [1], Xiaoming Pan [1], Huaming Li [1] and Dapeng Peng [1,2,3,4,*]

[1] National Reference Laboratory of Veterinary Drug Residues (HZAU) and MOA Key Laboratory for Detection of Veterinary Drug Residues, Huazhong Agricultural University, Wuhan 430070, China; zhanglinwei9910@163.com (L.Z.); yinxy73251@163.com (X.Y.); tiep990212@163.com (H.Y.); w793653717@163.com (H.W.); hanshiyun2022@163.com (S.H.); pxiaoming2596@163.com (X.P.); lihm9915@163.com (H.L.)

[2] State Key Laboratory of Agricultural Microbiology, Huazhong Agricultural University, Wuhan 430070, China

[3] Shenzhen Institute of Nutrition and Health, Huazhong Agricultural University, Wuhan 430070, China

[4] Shenzhen Branch, Guangdong Laboratory for Lingnan Modern Agriculture, Genome Analysis Laboratory of the Ministry of Agriculture, Agricultural Genomics Institute at Shenzhen, Chinese Academy of Agricultural Sciences, Shenzhen 518000, China

[*] Correspondence: pengdapeng@mail.hzau.edu.cn; Tel.: +86-27-8728-7165; Fax: +86-27-8767-2232

[†] These authors contributed equally to this work.

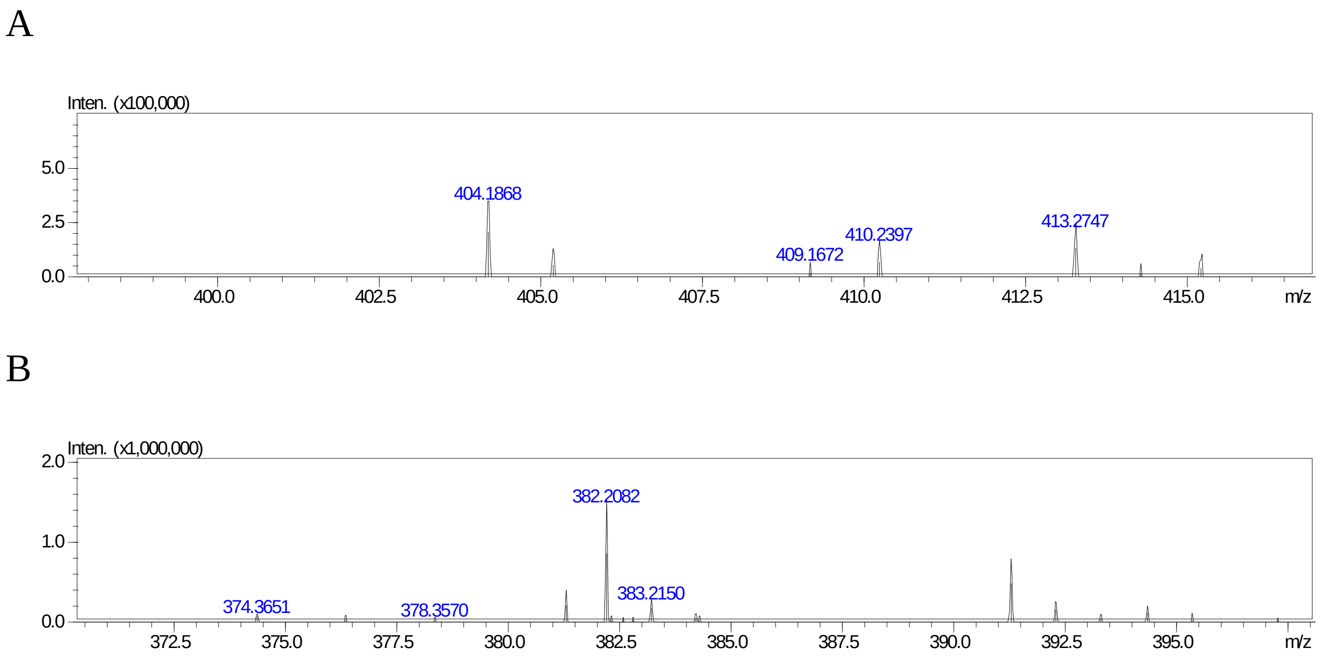

Figure S1. PR-SC mass spectrometry (**A**: [M+Na]+ and **B**: [M+H]+).

Table S1. Titer and inhibition rate of serum PY and PR antibody in mice.

Mice No.	Serum dilution factor（1：X）	Inhibition rate (B/B0)[a]
PRA1	4000	32%
PRA2	4000	31%
PRA3	2000	48%
PRA4	4000	58%

[a] The above inhibition rates were calculated at a concentration of 250 μg/L PR.

Table S2. PR cell antibody titers and inhibition rates.

Cell Line No.	Antibody titer（1：X）	Inhibition rate [a]
PR-114	80	42%
PR-112	40	45%
PR-121	80	53%
PR-142	80	53%

[a] The above inhibition rates were calculated at a concentration of 50 μg/L PR.

Table S3. PR/114 antibody array titration.

Antibody concentration（μg/mL）	Coated antigen concentration（μg/mL）					
	**1**	**0.5**	**0.25**	**0.125**	**0.0625**	**0.03125**
2	3.132	3.091	2.888	2.642	2.302	1.887
1	2.969	2.684	2.459	2.212	1.905	1.487
0.5	2.386	2.294	1.966	1.851	1.000	0.963
0.25	1.714	1.561	1.422	1.241	1.011	0.695
0.125	1.150	1.097	1.086	0.944	0.838	0.666
0.0625	0.863	0.881	0.806	0.788	0.701	0.599

Table S4. Screening for PR/114 antibody and PR-SC-OVA concentrations.

Coated antigen concentration（μg/mL）	Antibody concentration（μg/mL）	IC_{50}（μg/L）
0.25	0.5	47.06
0.125	0.5	35.41
0.0625	1	31.47

Table S5. CV of PR/114 antibody ic-ELISA standard curve.

PR(μg/L)	Measured value (C±SD, μg/L)	Coefficient of Variation (%) (CV%, n=5)	measured value (C±SD, μg/L)	Coefficient of Variation (%) (CV%, n=25)
6.25	5.92±0.78	13.1	6.18±0.26	4.2
	6.03±0.82	13.6		
	6.28±0.97	15.4		
	6.02±0.78	12.9		
	6.65±0.74	11.1		
12.50	13.52±2.07	15.3	13.35±1.04	7.7
	12.02±1.87	15.5		
	12.34±1.94	15.7		
	14.07±1.06	7.5		
	14.79±1.84	12.4		
25.00	27.52±2.04	7.4	27.34±0.50	1.8
	27.98±1.94	6.9		
	27.40±1.60	5.8		
	27.34±2.01	7.3		
	26.45±1.01	3.8		
50.00	56.21±4.25	7.5	52.69±3.13	5.9
	52.05±2.22	4.2		
	56.39±4.39	7.7		
	50.01±5.16	10.3		
	48.78±1.07	2.1		
100.00	89.94±6.12	6.8	87.85±1.21	1.3
	87.33±5.42	6.2		
	88.38±6.38	7.2		
	86.56±2.93	3.3		
	87.01±5.57	6.4		

华中农业大学实验动物中心动物实验伦理审查表

The Tab of Animal Experimental Ethical Inspection of Laboratory Animal Centre, Huazhong Agriculture University

伦理编号(ID Number)：HZAUMO-2022-0219

申请人填写的相关信息 (Related information filled by applicant)	申请单位(Name of organization)		华中农业大学兽药GLP实验室				
	项目负责人姓名(Name of project director)		彭大鹏		项目负责人职称(Professional title)		教授
	是否本单位做实验		否		实验名称(Experiment title)		丙草胺单克隆抗体制备
	项目经费来源(Project sources)		非国家项目				
	实验目的(Aim of experiment)		获得能够产生针对吡蚜酮抗体的小鼠，并获得抗体				
	实验动物使用许可证号(Number of the using of Laboratory Animal)		SYXK（鄂）2019-0044				
	拟进动物情况	动物来源(Source of animal)	三峡大学实验动物中心				
		品种品系(Species or strain)	小鼠、BALB/c	等级(Grade)	SPF	规格(Specifications)	小鼠5-6周龄、小鼠
		数量(Number)	16只（♀ 16 只；♂ 0只）		申请日期(Application date)	2022-12-05	
		进驻日期(Entering date)	2022-12-05		结束日期(Ending date)	2023-03-30	
	实验要点，包括实验方法、观测指标、实验结束后处死动物的方法等 (Outline of experiments, experimental methods, observational index, executing animal method, et. al)						
	该研究本着实验动物福利和伦理的原则，优化设计方案，严格计划动物需要数量。计划需要16只雌性BALB/c小鼠。试验前，在试验条件下观察3-5天，然后开始实验。小鼠背部皮下免疫吡蚜酮抗原，免疫程序采用一次基础免疫及数次加强免疫，加强免疫腹腔注射免疫原，二免之后采血测定抗体效价，待血清效价达到目标后，将小鼠脱颈椎处死，采取脾脏。小鼠尸体交给实验室统一进行无害化处理。						
	申请人签名 (Signature of applicant)		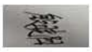		联系电话 (Telephone)		15090373251
	项目负责人签名 (Signature of Project director)		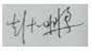		联系电话 (Telephone)		13657290856

声明(Statement)：

我将自觉遵守实验动物福利伦理原则，随时接受委员会的监督与检查，如违反规定，自愿接受处罚。

(I will conscientiously abide by the ethical principles of animal welfare, accept the supervision and inspection of the committee at any time, and voluntarily accept the punishment if any infringement.)

项目负责人签名(Signature of Project director)： 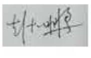

项目执行人签章(Signature of Project implementation)： 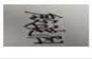

<table>
<tr><td>审查依据
(Inspectionconte nts)</td><td colspan="3">1.该项目是否必须用实验动物进行实验，即能否用计算机模拟、细胞培养等非生命方法替代动物或用低等动物替代高等动物进行实验?
(Does laboratory animal must be used in the project? Could other methods such as computer simulation, cell cultivation or using the low-grade animal instead of the high-grade animal?)
2.表中所填申请人资格和所用动物的品种品系、质量等级、规格是否合适，能否通过改良设计方案或用高质量的动物来减少所用动物的数量?
(Are the qualification of applicant，species or strain, grade and specifications of animals suitable? Could the quantity of animals be reduced by improving the study design or using high quality animals?)
3.能否通过改进实验方法、调整实验观测指标、改良处死动物的方法，来优化实验方案、善待动物?
(Could the study design and animal treatment be refined by ameliorating experimental method, adjusting observational index, executing animal method?)</td></tr>
<tr><td rowspan="3">专家审查
(Professor inspection)</td><td>专家意见
(Suggestion of Professor)</td><td>专家签名
(Signature of Professor)</td><td>日期
(Date)</td></tr>
<tr><td>同意</td><td>王旭</td><td>2022年 12 月 23日</td></tr>
<tr><td>同意</td><td>陶攀</td><td>2022年 12 月 15日</td></tr>
<tr><td colspan="4">科学伦理委员会负责人签名(Signature of Ethics Committee Director)：
赵书红
2022年 12 月 24日</td></tr>
<tr><td>审查结果
(是否同意申请人的实验方案)
(Results of inspection)</td><td colspan="3">伦理委员会意见
(Attitude of the Ethics Committee)：
同意
华中农业大学科学伦理委员会
2022年 12 月 24日</td></tr>
</table>
